# The American Medical Association

**Published:** 1849-06

**Authors:** 


### THE WESTERN JOURNAL.

THIRD SERIES.—VOL. III.—NO. VI.

LOUISVILLE, JUNE 1, 1849.

### THE AMERICAN MEDICAL ASSOCIATION.

The American Medical Association held its second annual meeting at Boston on the first day of May, and adjourned on Friday the 4th, after an interesting session of four days. The delegates were welcomed to the city by Dr. Warren in a neat address, which was replied to by several members of the Association. The officers appointed for the following year are as follows:

For President—Dr. John C. Warren, of Massachusetts.

For Vice Presidents—Dr. J. P. Harrison, of Ohio; Dr. H. H. Maguire, of Virginia; Dr. A. Flint, of New York; Dr. R. S. Stewart, of Maryland.

For Secretaries—Dr. A. Stille, of Pennsylvania; Dr. H. I. Bowditch, of Massachusetts.

For Treasurer—Dr. Isaac Hays, of Pennsylvania.

The next meeting will be held in Cincinnati. Of the late meeting, the editor of the Boston Medical and Surgical Journal says, that it was "eminently characterized by harmony, order, and good fellowship." The delegates alone numbered about 450, and were from twenty-two States of the union. The communications and reports made during the session are spoken of as able and important. As at the former meetings, the subject which called forth most discussion was that of medical education, in reference to which the following resolutions were adopted:

"Resolved, That the Association reiterate their approval of the resolutions, in reference to medical education, adopted by the Convention which met in Philadelphia in May, 1847, and contained in pages 73 and 74 of the published proceedings of that Convention.

"Resolved, That the attention of medical colleges be again directed to the resolutions of the Committee on Preliminary Education, adopted by the Medical Convention of 1847, and that they be advised to require from the students, in all instances, certificates of due preliminary acquirement prior to graduation.

"Resolved, That physicians generally, throughout the Union, be advised and requested to requre of those wishing to become their pupils, evidence of a proper general education, before admission into their offices.

"Resolved, That this Association does not sanction or recognise "college clinics" as substitutes for hospital clinical instruction, and that the medical colleges be again advised to insist, in all instances when it is practicable, on the regular attendance of their pupils, during a period of six months, upon the treatment of patients in a well-conducted hospital, or other suitable institution devoted to the reception and cure of the sick.

"Resolved, That in accordance with a resolution of the American Medical Association adopted May 4th, 1847, it is earnestly recommended to the physicians of those States in which State Medical Societies do not exist, that they take measures to organize them before the next annual meeting of this Association.

"Resolved, That the State Societies be recommended, after they shall have been organized, to recognize as regular practitioners none who have not obtained a degree in medicine, or a license from some regular medical body, obtained after due examination.

"Resolved, That this Convention recommend to the various schools of medicine to meet at Cincinnati before the next meeting of this Association, and present a plan for elevating the standard of practical education."

